# Electrochemical performance of Ni_3_V_2_O_8_/rGO composites for supercapacitor applications

**DOI:** 10.1039/d6ra05505b

**Published:** 2026-10-20

**Authors:** B. Kabilan, Manikandan Ayyar, S. Sakthivel, Rajendra P. Patil, V. Mohanavel, S. Rajendran, Shanmugapriya D., Tahseen Kamal, Anish Khan, Hadi M. Marwani, Yasmeen G. Abou El-Reash, Hany Koheil, Basem E. Keshta

**Affiliations:** a Department of Chemistry, Karpagam Academy of Higher Education Coimbatore-641021 Tamil Nadu India manikandan.frsc@gmail.com; b Centre for Material Chemistry, Karpagam Academy of Higher Education Coimbatore-641021 Tamil Nadu India; c Department of Chemistry M. H. Shinde Mahavidyalaya, Tisangi Kolhapur 416 206 MH India; d Centre for Sustainable Materials Research, Department of Mechanical Engineering, Academy of Maritime Education and Training (AMET), Deemed to Be University Kanathur Chennai 603112 Tamil Nadu India; e International Center of Nanotechnology and Functional Materials, Instituto de Alta Investigaci, ó, n, Universidad de Tarapacá Arica-1000000 Chile; f Vicerrectoría de Investigación y Postgrado, Universidad de La Serena Código Postal: 1700000 Chile; g Center of Excellence for Advanced Materials Research, King Abdulaziz University Jeddah Saudi Arabia; h Department of Chemistry, College of Science, Imam Mohammad Ibn Saud Islamic University (IMSIU) P.O. Box 90950 Riyadh 11623 Saudi Arabia; i Physics and Engineering Mathematics Department, Faculty of Engineering, Kafr Elsheikh University Kafr Elsheikh 33516 Egypt; j Chemistry Department, Faculty of Science, Tanta University Tanta 31512 Egypt basem.keshta@science.tanta.edu.eg; k Chemistry Department, Faculty of Science, King Abdulaziz University Jeddah-21589 Saudi Arabia

## Abstract

Hydrothermally synthesized Ni_3_V_2_O_8_ (NV) and Ni_3_V_2_O_8_–reduced graphene oxide (NV–R) nanocomposites offer promising electrode materials for high-performance supercapacitors. The crystal structure and the phase purity of the composites were confirmed by X-ray diffraction (XRD). Fourier transform infrared (FTIR) spectroscopy was confirmed the characteristic of M–O (M = Ni, V) vibrations. Field-emission scanning electron microscopy (FE-SEM) and high-resolution transmission electron microscopy (HR-TEM) images confirmed that well-integrated Ni_3_V_2_O_8_ nanoparticles were uniformly distributed on the rGO nanosheets. Cyclic voltammetry (CV), galvanostatic charge/discharge (GCD) and electrochemical impedance spectroscopy (EIS) were used to assess the electrochemical performance. The intimate contact between rGO and Ni_3_V_2_O_8_ is consistent with rapid electron transport, good electrical conductivity, and a large electrochemically active surface area. Therefore, at a current density of 1 A g^−1^, the NV–R electrode achieved a high specific capacitance of 494 F g^−1^, which is larger than that of the NV electrode (264 F g^−1^). In addition, the assembled asymmetric supercapacitor device exhibited a specific capacitance of 81 F g^−1^, a specific energy density of 22.05 Wh kg^−1^, and a power density of 4177 W kg^−1^, maintaining 86.76% of the initial capacitance after 5000 charge–discharge cycles. The findings show that the NV–R/NF electrode, which is grown *in situ*, exhibits electrochemical performance and could be one of the ideal candidates for next-generation high-performance applications in supercapacitors.

## Abstract 2

Ni_3_V_2_O_8_/rGO nanocomposites grown hydrothermally on Ni foam deliver 494 F g^−1^ at 1 A g^−1^; the NV–R//AC device gives 22.05 Wh kg^−1^ at 4177 W kg^−1^ with 86.76% retention after 5000 cycles.

## Graphical abstract

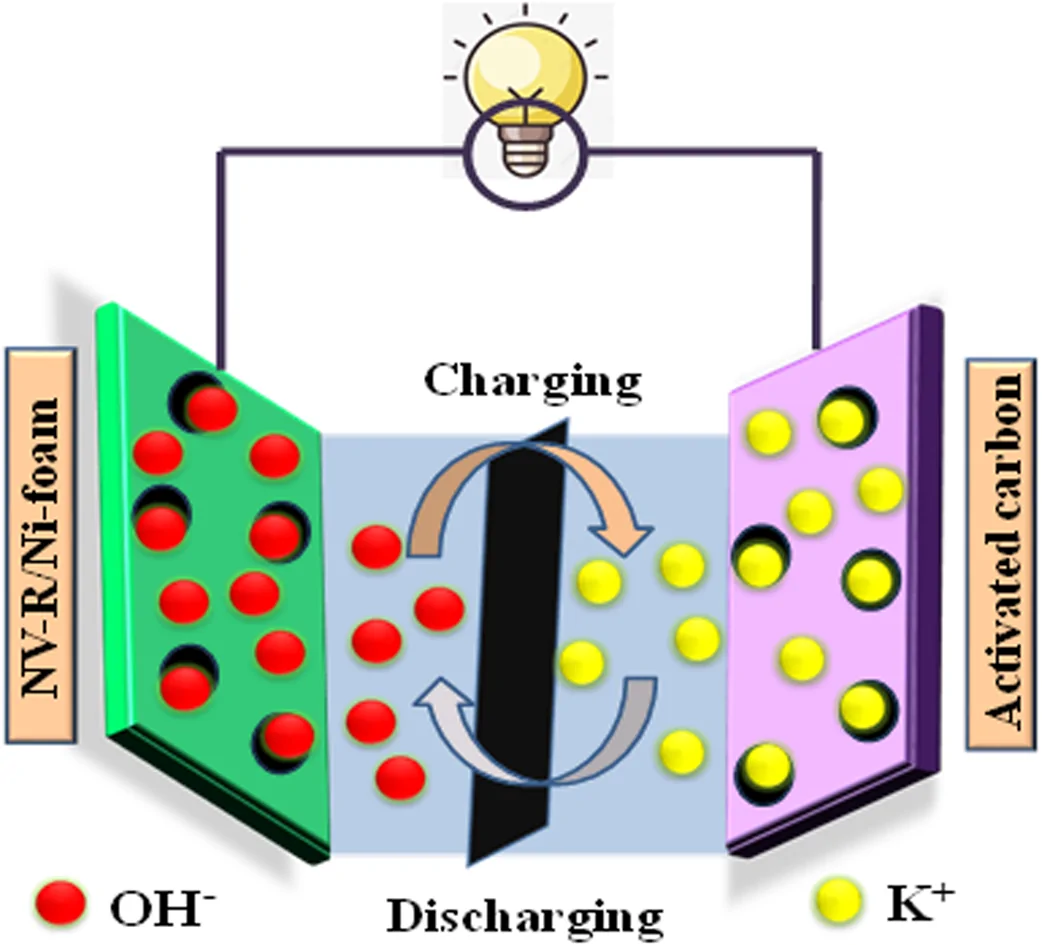

## 1. Introduction

For many decades, conventional energy sources have satisfied almost all human needs, but they also contribute to environmental pollution by releasing heat and greenhouse gases and causing other problems. Additionally, as the population grows and the demand for electricity increases, available resources are rapidly declining, making it difficult to address this issue. Hence, most researchers have focused on novel, eco-friendly sources that are independent of the season and time.^1,2^ Systems for converting chemical energy into electricity *via* electrochemical energy storage (EES) have become a competitive alternative to conventional sources in recent years.^3^ In general, the capacitors and batteries possess high power and enormous energy density, respectively. Moreover, a “supercapacitor” (SC) is a device that operates between batteries and capacitors. High diffusion kinetics, power supply, excellent recyclability, long lifespan, and low maintenance are among its distinctive features.^4^ High power and energy densities are crucial in consumer hybrid electric cars, portable gadgets, and medical devices, all of which make extensive use of SCs. SCs are of two types: pseudocapacitors and electric double-layer capacitors (EDLCs), which depend on non-faradaic processes and quick redox reactions.^5^ EDLCs show little promise for energy storage applications, because they have high energy density. The pseudocapacitors utilized in energy storage systems have received a lot of attention.^6^ Transition metal oxides (TMOs) such as Bi_2_O_3_, V_2_O_5_, Co_3_O_4_, NiO, RuO_2_, and MnO_2_ are frequently used as electrodes in both symmetric and asymmetric designs. However, they provide low energy density and poor cycling stability.^7,8^ To overcome these challenges, carbon-based materials are combined to prepare an electrode for energy storage applications. Because of their wide surface area, outstanding stability, strong conductivity, tunable pore size, and exceptional mechanical strength, they have attracted attention recently.^9,10^ Graphitic and non-graphitic carbons are the two categories of carbons. Graphite may be exfoliated to create graphene, a 2D material with a densely packed conjugated hexagonal lattice. Like other 2D materials, GO has a large specific surface area, making it ideal for maximizing electrochemically active sites. Reduced graphene oxide (rGO) is usually replaced by graphene.^11^ It may interact physically with porous carbon materials, due to its extensive ring structure, which is similar to graphene. Furthermore, the creation of solid-state devices for supercapacitors are facilitated by the presence of many functional groups that chemically interact with conducting polymers. Moreover, rGO provides a larger number of active areas.^12,13^

Extensive research has focused on enhancing the electrochemical performance of binary metal oxides (A_*x*_B_*y*_O_*z*_), due to their changeable electronic architecture, various oxidation states, and excellent chemical/thermal stability.^14^ The incorporation of carbonaceous materials such as rGO with metal oxides to enhance the composite functionality for energy-related applications. Recently, nickel-based metal oxide electrode system shows a considerable potential for advanced energy storage, with substitution strategies further tuning the electrochemical response. Generally, ABO_4_, AB_2_O_4_, and A_3_B_2_O_8_ types of metal oxides with low (A) and high (B) oxidation states. Among them, metal vanadates (M_3_V_2_O_8_, M = Ni) with a Kagome-staircase structure, like nickel vanadate, have the potential to perform very well in supercapacitor applications.^15^ Besides the electrode, the electrolyte is another key component in the supercapacitors. KOH aqueous electrolytes are considered effective, because of their high ionic concentration and a low resistance. Higher KOH concentrations lead to higher ionic conductivity, which promotes greater ion transport between the electrolyte and the electrode. They also reduce the diffusion resistance of the materials, which facilitates electrochemical processes.^16,17^ Wei-Bin Zhang *et al.* synthesized Ni_3_V_2_O_8_/NiO composites and they have achieved the capacitance value of 2068 F g^−1^.^18^ Lakshmana Naik *et al.* synthesized Ni_3_V_2_O_8_ for supercapacitor applications, and found the capacitance of 398 F g^−1^.^19^ Vishnukumar *et al.* reported that the NiO/Ni_3_V_2_O_8_ as the supercapacitor and achieved the capacitance value of 653 F g^−1^ at 1 A g^−1^.^20^ Shraddha B. Bhosale *et al.* reported that the Ni_3_V_2_O_8_ cathodes resulted a capacitance of 692 F g^−1^.^21^ Chandra Sekhar *et al.* reported the chain-like nickel pyro-vanadate and they achieved a specific capacity of 65 µA h cm^−2^.^22^ The performance of Ni_3_V_2_O_8_ electrodes, especially in EES applications, is related to ion transport parameters and electronic conductivity. Two-dimensional (2D) electrode materials grown directly on a porous-structured nickel foam (NF), which offers a high surface-to-volume ratio, have attracted much interest. By growing Ni_3_V_2_O_8_ directly on the NF with negligible interfacial resistance and the ion transfer can be improved.^23^ Because of its multiple oxidation states, Ni_3_V_2_O_8_ has potential for high theoretical capacitance as an electrode material for supercapacitors, but its practical application is limited by the fact that it is poorly conducting and also structural degradation during repeated redox cycles, and a systematic understanding of the modulation of its charge-transport and redox kinetics by carbon hybridization is still incomplete. The novelty of this work is the ability to combine Ni_3_V_2_O_8_ with conductive rGO to simultaneously increase the electrical conductivity, suppress the degradation of the structure with repeated cycles, and encourage synergistic faradaic and electric double-layer charge-storage mechanisms to improve the electrochemical performance.

To overcome this challenges, here we developed a Ni_3_V_2_O_8_/rGO composite that combines the structural and electronic functions of rGO as a conductive scaffold to reduce the intrinsic resistivity of Ni_3_V_2_O_8_ and the anchoring effect as a structural component to inhibit the restacking of graphene and make more electroactive sites available. Such improvement in capacitive performance is due to the synergistic effect on the charge storage mechanism of Ni_3_V_2_O_8_, which provides the faradaic charge storage mechanism with reversible Ni^2+^/Ni^3+^ and V^4+^/V^5+^ redox processes, and the rGO framework, which provides the electric double-layer capacitance mechanism at the electrode/electrolyte interface and enhances the charge storage capacity, with the additional benefit of rapid electron transfer.

The current work focuses on the synthesis and electrochemical performance of NV and NV–R NCs on the NF substrate. The single-step hydrothermal technique was used to produce NV and NV–R NCs and to bind the nickel foam in a reaction chamber. The morphological behaviour, surface analysis, and structural investigation of the prepared NV and NV–R NCS were carefully assessed. EIS, CV, and GCD were used to examine the electrochemical properties of pure and composite materials. Furthermore, the fabricated devices, which do not contain toxic materials, can be a potential alternative to energy storage devices.

## 2. Materials and methods

### 2.1 Materials

All the chemicals used in this study, nickel nitrate hexahydrate (Ni(NO_3_)_2_·6H_2_O), ammonium metavanadate (NH_4_VO_3_), potassium hydroxide (KOH), urea (CO(NH_2_)_2_), reduced graphene oxide (rGO), and ethanol, were of analytical reagent (AR) grade with purity ≥99% (KOH, ≥85%) and were purchased from Merck and used as received without further purification.

### 2.2 Preparation of Ni_3_V_2_O_8_ NPs

All precursors are analytical-grade used to prepare NV and NV–R materials by hydrothermal method. Initially, 0.1 M Ni(NO_3_)_2_·6H_2_O, 0.1 M NH_4_VO_3_, and 0.1 M CO(NH_2_)_2_ were dispersed in 70 mL of DD water, and the mixture was stirred until a homogeneous solution was obtained. This process is illustrated in Fig. 1. Then, an NF substrate was immersed in the solution. The solution was transferred into a 100 mL autoclave and placed in a vacuum oven for 12 h at 180 °C. The autoclave was allowed to reach room temperature. The resulting sample was filtered, washed with DD water and ethanol and dried at 60 °C, and the product was annealed for 4 h at 450 °C in a muffle furnace to obtain the NV sample. The NV–R was synthesized by the same route with 20 mg rGO was added.

**Fig. 1:**
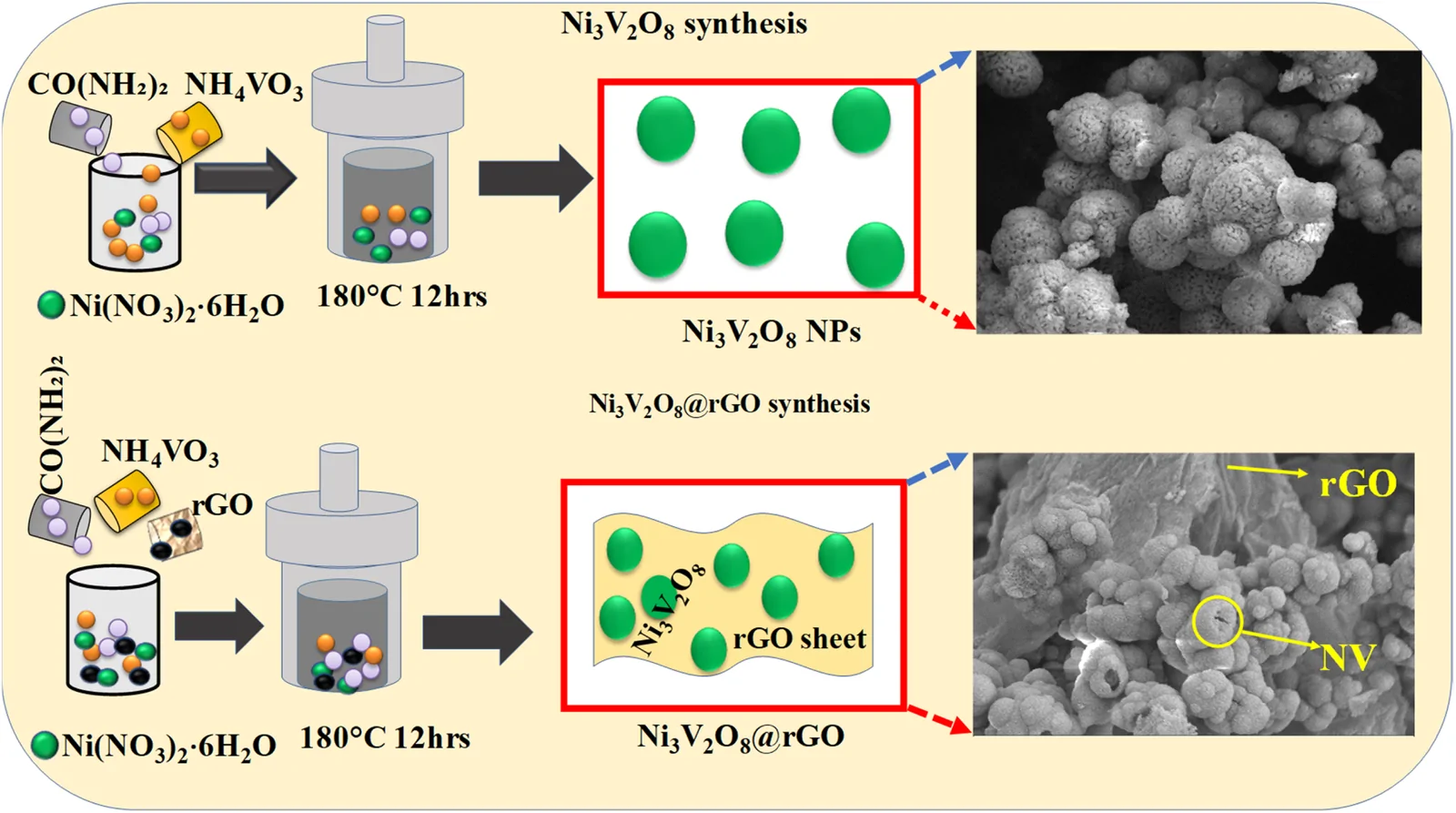
Schematic diagram of the synthesis procedure of NV and NV–R samples.

### 2.3 Materials characterization

XRD patterns were recorded using a Bruker D8 diffractometer with Cu-Kα radiation (*λ* = 1.5406 Å) over a 2*θ* range of 10°–80° at a scan rate of 5°/min. FTIR spectra were recorded using a PerkinElmer spectrometer in the wavenumber of 400–4000 cm^−1^ range in ATR mode. SEM images were acquired using a ZEISS Gemini SEM 300 field-emission scanning electron microscope (FE-SEM) operated at accelerating voltages of 5 to 10 kV. HR-TEM and SAED analyses were performed using a JEM-2100 Plus operating at 200 kV. The XPS spectra were recorded using a PHI Versa Probe III system with a monochromatic Al Kα X-ray source (1486.6 eV), and all electrochemical measurements (CV, GCD, EIS) were performed using a CHI 660E electrochemical workstation in a three-electrode configuration using a 6 M KOH electrolyte, a Pt counter electrode, and a Hg/HgO reference electrode, with CV scan rates of 10–200 mV s^−1^, a potential window of 0–0.5 V (three-electrode) and 0–1.5 V (two-electrode ASCs), and EIS recorded over 100 kHz–0.1 Hz.

### 2.4 Electrode fabrication and supercapacitor applications

The NV and NV–R composites were grown directly on the NF (2 × 2 cm^2^) as current collector; the architecture of directly grown composites was binder-free and without the addition of a conductive additive, leading to close contact between the active material and the current collector, which reduced the interface resistance. A platinum wire and a Hg/HgO electrode were used as the counter and reference electrodes, respectively. For comparison, bare NF, NF/rGO, and a physically mixed (PM) electrode as control NV–R/PM electrode were also prepared. The NF/rGO control electrode was fabricated by doctor-blade coating an rGO slurry (8 : 1 : 1) onto a pre-cleaned NF. The NV–R/PM control electrode was prepared by physically mixing pre-synthesized NV and rGO powders in a mass ratio of 9 : 1 using an agate mortar and pestle until a homogeneous mixture was obtained. The resulting powder was then dispersed into a slurry containing the active material, conductive carbon black, and PVDF binder in a weight ratio of 8 : 1 : 1. The slurry was uniformly coated on the NF (1 × 1 cm^2^) using the doctor-blade method and dried under the same conditions as those used for the NF/rGO electrode. The active material loading for the three-electrode electrochemical measurements at 3 mg for the NV electrode and 3.7 mg for the NV–R electrode on the NF (1 × 1 cm^2^).1
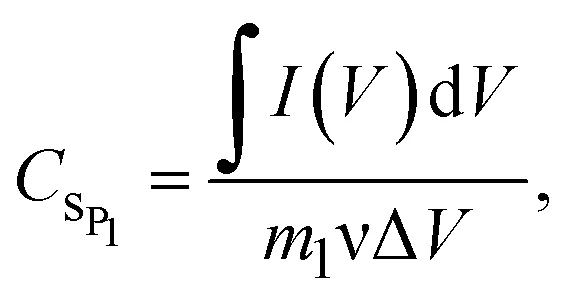
2
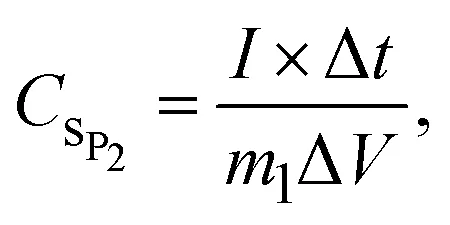
where *m* (mg) is the material mass, *I* (A) is the discharge current, Δ*V*(V) is the potential window, and Δ*t* (s) is the discharging time.

The energy density (*E*_d_) and power density (*P*_d_) of the device were calculated using the following equation:3
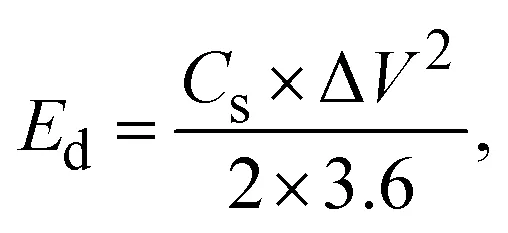
4
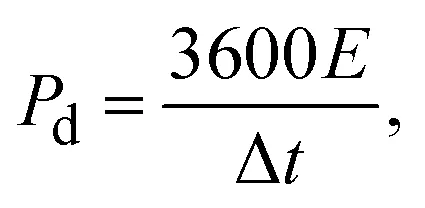
where *V* is the potential and *t* is the discharging time.

The coulombic efficiency was determined using the following formula:5
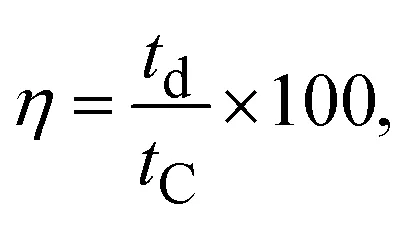
where *t*_C_ and *t*_d_ represent the charging and discharging times, respectively. The electrochemical performance was evaluated over a potential window of 0–1.5 V. The two-electrode asymmetric supercapacitor (ASC) device employed an NV–R composite as the positive electrode and activated carbon (AC) as the negative electrode. A Whatman filter paper impregnated with a PVA/6 M KOH gel electrolyte. To achieve the optimal charge storage performance, the mass loadings of the positive and negative electrodes were balanced according to the charge-balance principle (*q*+ = *q*−), using the following equation:6
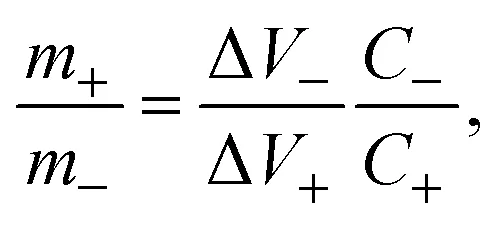
where *C*_+_ and *C*_−_ are the specific capacitances, and Δ*V*_+_ and Δ*V*_−_ are the potential windows of the positive and negative electrodes, respectively. The calculated mass ratio of the positive and negative electrodes was approximately 1 : 1. The total active material mass of the assembled cell was 4.6 mg on the NV–rGO positive electrode and 4.4 mg on the AC negative electrode.

## 3. Results and discussions

### 3.1 XRD and FT-IR analysis

XRD analysis was performed to analyze the phase composition of the synthesized materials. The observed patterns are shown in Fig. 2(a). The XRD peaks of Ni_3_V_2_O_8_ (NV) are matched with 2*θ* values of 30.91°, 35.90°, 43.84°, 58.52°, and 63.69°, which correspond to the *hkl* plane values of (131), (221), (004), (115), and (244), respectively,^24,25^ which match the JCPDS card number 01-070-1394 and orthorhombic crystal structure.^26^ The XRD pattern of the NV–R composite is dominated by the diffraction peaks of crystalline NV. Weak reflections corresponding to rGO were also observed, which were attributed to the low content and poor crystallinity of rGO. A minor shift in the NV–R diffraction peaks relative to pristine NV was also observed, which attributed to slight lattice strain in the near-surface region of NV induced by its intimate contact with the rGO sheets during growth, together with minor interactions between oxygen-containing functional groups on rGO and the NV lattice. Similarly, the rGO was observed at 2*θ* angles of 23.20° and 43.14°, which match the (002) and (100) crystalline planes, respectively.^27–29^ The Scherrer equation was used to determine the crystalline size of NV and NV–R NCs as follows:

7 *D* = *Kλ*/(*β* cos *θ*),

where *λ* is the wavelength of X-ray, *K* is the shape factor, *θ* is the diffraction angle, and *β* is the FWHM. The crystalline size of the prepared NV and NV–R samples are 26 and 22.05 nm, respectively.

**Fig. 2:**
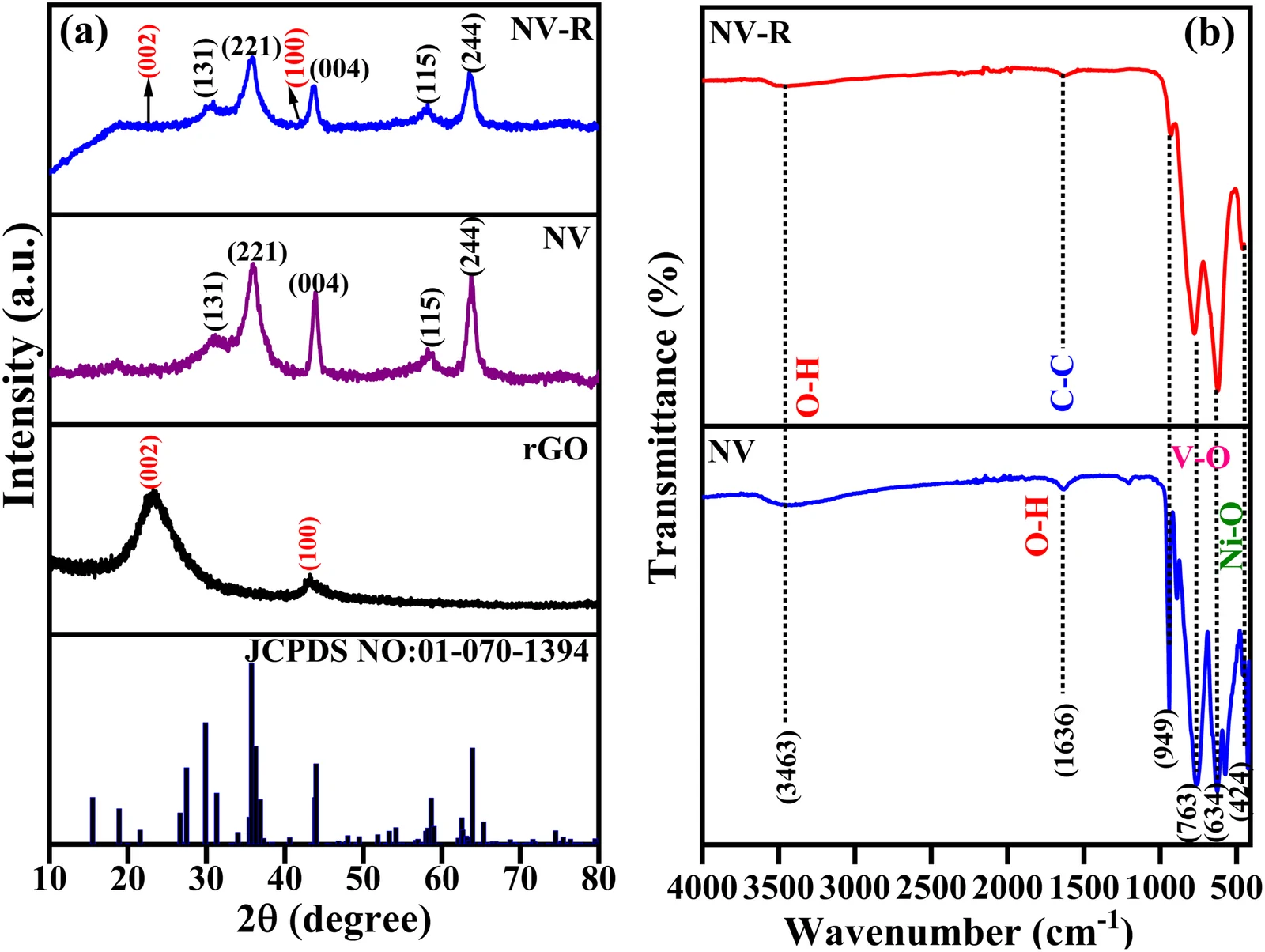
(a) XRD pattern of NV, rGO and NV–R NCs. (b) FT-IR spectra of NV and NV–R NCs.

The functional groups of the samples were identified by FT-IR spectroscopy. The recorded spectrum is shown in Fig. 2(b). The hydroxyl (O–H) vibrations are observed at 3463 cm^−1^, which can enhance ion transport and electrolyte wettability during the electrochemical process. The stretching vibrations (C–C) of rGO were seen at 1636 cm^−1^.^30,31^ The C–C stretching vibration at 1636 cm^−1^ indicates the presence of carbonaceous species associated with rGO in the composite. The V–O vibrations appear at 949, 763, and 634 cm^−1^.^32^ Vanadium acts as an active redox centre, contributing to pseudo-capacitance, due to its various oxidation states. The peaks at 424 cm^−1^ represent the Ni–O bonds.^33^

### 3.2 Raman analysis

The Raman spectra of the pristine NV and the NV–R composite are displayed in Fig. 3. NV and NV–R display a major peak at around 815–865 cm^−1^, which is assigned to the symmetric stretching vibration of the V–O in the VO_4_ tetrahedra of NV.^34^ This is a confirmation of the successful formation of the NV phase. A weaker band at 925 cm^−1^ is attributed to the stretching vibration of the V–O in the VO_4_ units.^35^ This band is less pronounced in the NV–R composite, suggesting that NV interacts with the rGO sheets. The primary Raman peak in the NV–R composite is slightly shifted in width and has a slight reduction in its intensity when compared to the pristine NV. The reason is mainly due to the incorporation of rGO into the material, which causes structural disorder and enhances the interaction between NV and the conductive carbon network.^36^ This kind of interaction reflects enhanced physical contact between NV and the conductive carbon network, without altering the crystal structure of NV. The D (1350 cm^−1^) and G (1580 cm^−1^) bands of rGO are not clearly seen in the recorded spectrum.^37^ This can be attributed to the rGO content is relatively low. However, the Raman results, in conjunction with the XRD, results, confirm the successful formation of the NV–R composite.

**Fig. 3:**
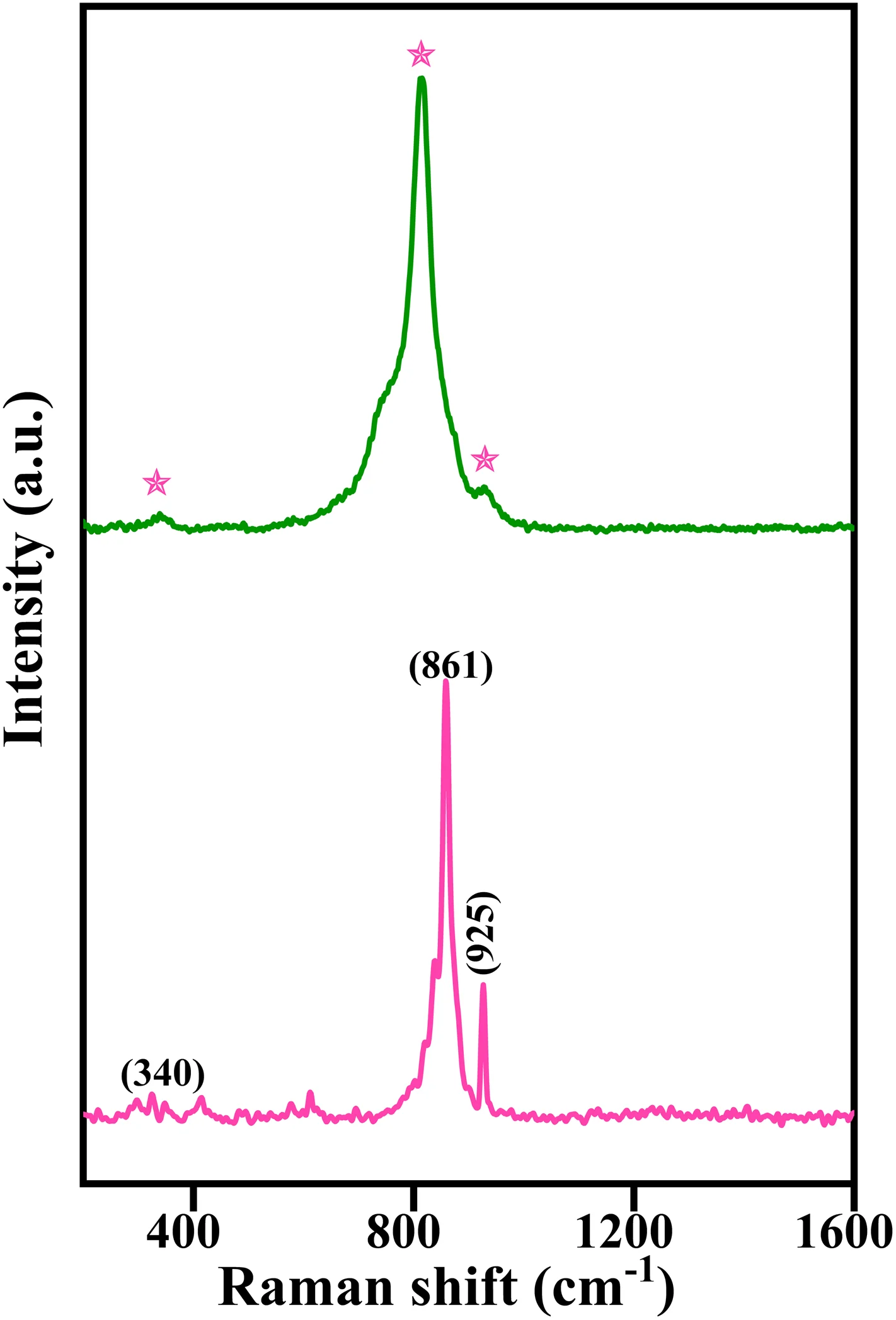
Raman analysis of NV and the NV–R composites.

### 3.3 SEM analysis

SEM analysis was performed to examine the surface morphology of the prepared NV, rGO and NV–R NCs are shown in Fig. 4. The SEM images revealed that the synthesized NV (Fig. 4(a and b)) possessed a cauliflower-like morphology. The SEM images of rGO, depicted in Fig. 4(c and d), revealed layered, sheet-like structures. The SEM images of NV–R NCs shown in Fig. 4(e and f) revealed two types of morphologies, including 2D sheet-like structures and cauliflower-shaped nanoparticles.^38^ The sheet morphologies were represented by the rGO sheets, and the NPs were attributed to NV. The SEM observations suggest that the NV nanoparticles are uniformly distributed on the rGO sheets, forming a well-dispersed composite morphology. The presence of rGO is expected to contribute to improved electrical conductivity and increased surface area.

**Fig. 4:**
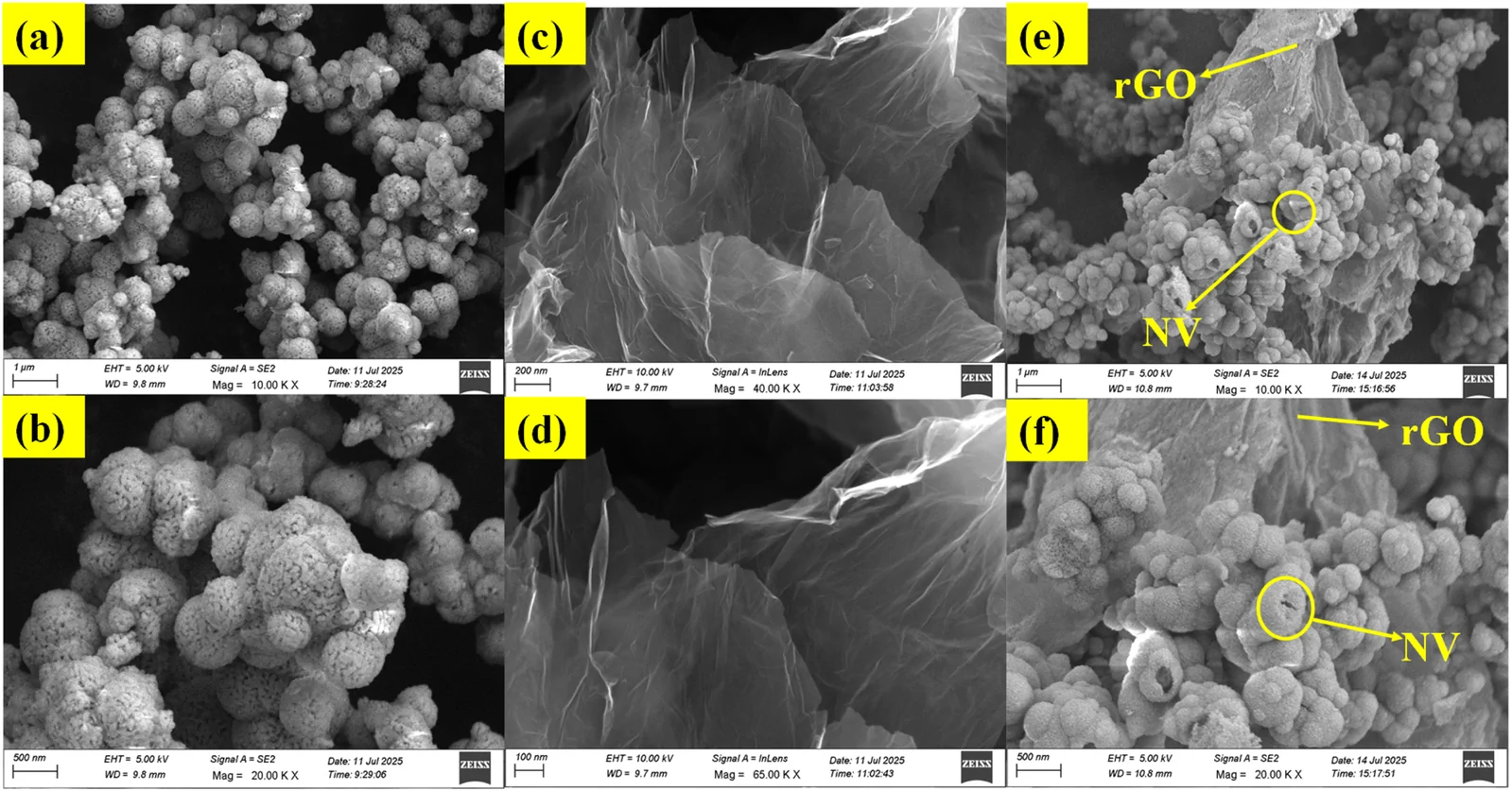
SEM images of (a and b) NV, (c and d) rGO, and (e and f) NV–R NCs.

The elemental composition of the pristine NV sample was analysed using energy-dispersive X-ray (EDX) spectroscopy, and the results are shown in Fig. 5(a–e). The elemental mapping images in Fig. 5(a–d) show the uniform distribution of Ni, V, and O across the sample, suggesting a uniform composition of the synthesized NV nanoparticles. According to the quantitative EDX analysis, the elemental weight percentages were 51.7, 34.1, and 14.2 wt% for Ni, V, and O, respectively. Fig. 5(e) shows the EDX spectrum of the synthesized sample, which exhibits characteristic peaks of Ni, V, and O, indicating the formation of NV without any contaminant elements. The results are consistent with the successful formation of the NV phase.

**Fig. 5:**
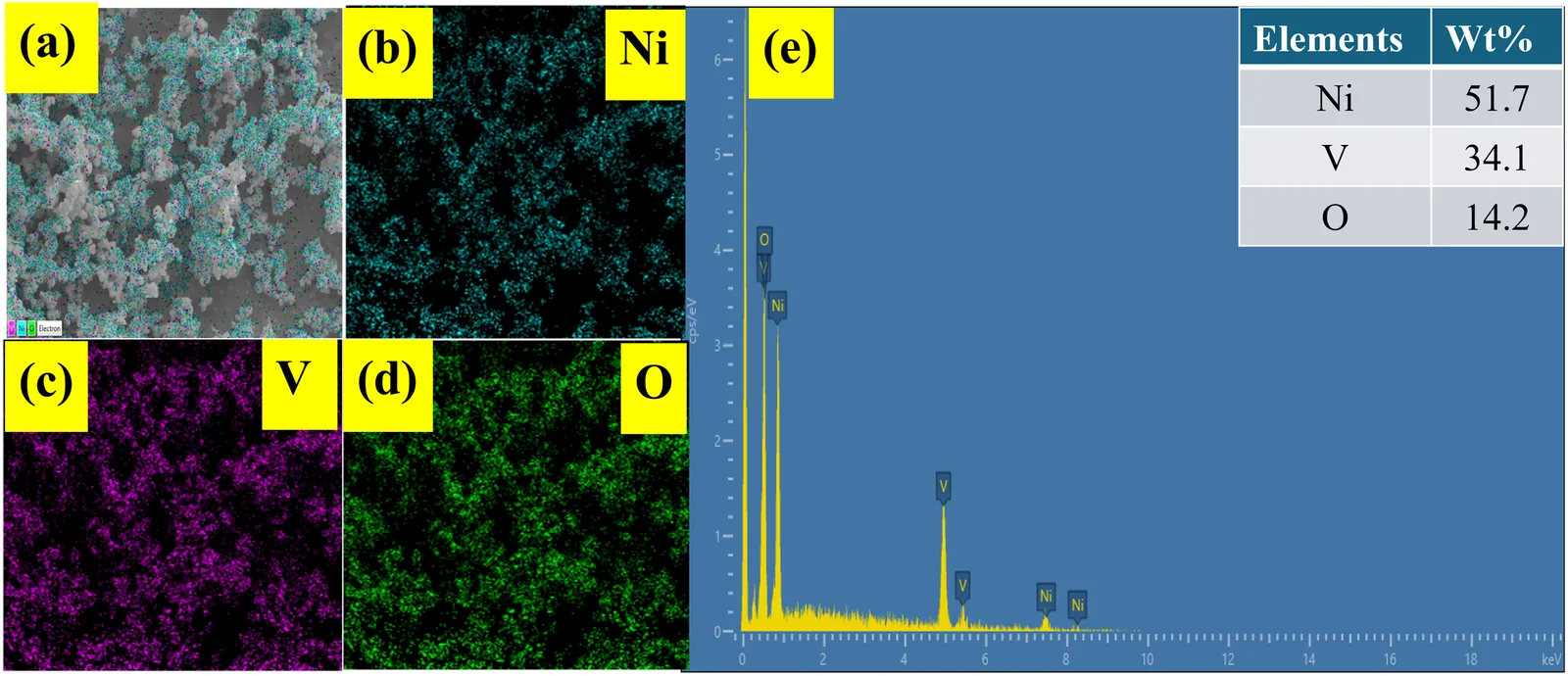
(a–d) Elemental mapping images and (e) EDX spectrum of NV.

The composition of the NV–R NCs was analyzed using energy-dispersive X-ray (EDX) spectroscopy. The mapping images are depicted in Fig. 6. Fig. 6(a) shows the EDX spectrum of the NV–R composite. According to the EDX spectrum, NV–R NCs contain Ni, V, C, and O peaks. The EDX spectra of NV–R NCs (Fig. 6(b–f)) show the Ni, V, C, and O peaks, indicating that the prepared NV–R NCs are composed of these elements. The elemental mapping confirms the uniform distribution of Ni, V, O, and C throughout the composite, indicating the coexistence of these elements.

**Fig. 6:**
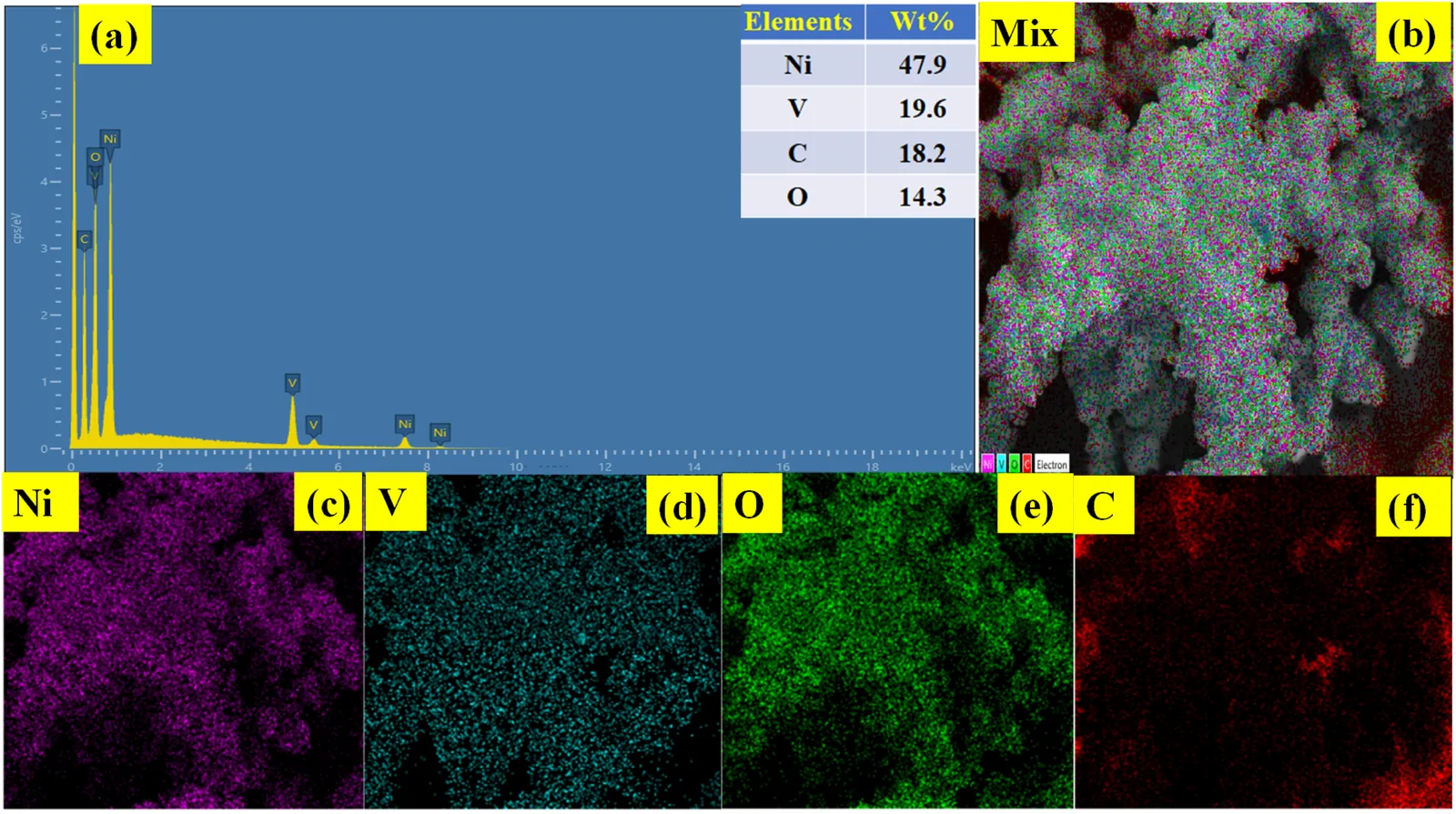
(a) EDX analysis of NV–R. (b–f) Elemental mapping images of Ni, V, O, and C.

### 3.4 XPS analysis

The XPS spectrum was utilized to analyze the oxidation state of the prepared NV–R NCs. XPS is used to identify specific compounds based on their unique binding energies and to determine the presence of elements. The presence of O, V, Ni, and C components in the prepared material is shown in Fig. 7(a). In Fig. 7(b), the spin–orbit peaks of Ni 2p_3/2_ and 2p_1/2_ are centred at 860 and 877 eV, respectively. There is also a satellite peak for Ni at 865.5 eV. However, both Ni^2+^ and Ni^3+^ are well assigned and present in NV–R.^39^ The high-resolution V 2p spectrum was denoted V^4+^ and V^5+^ components, as seen in Fig. 7(c). The peaks located at approximately 516.1 and 523.2 eV were assigned to V^4+^ (V 2p_3/2_ and V 2p_1/2_), whereas the peaks at approximately 516.7 and 524.5 eV correspond to V^5+^ (V 2p_3/2_ and V 2p_1/2_). The coexistence of V^4+^ and V^5+^ confirms the mixed valence state of vanadium in the NV–R sample, which can facilitate reversible V^5+^/V^4+^ redox reactions during electrochemical charge storage.^40^ The rGO with NV may result in the formation of carbon peaks in the C 1s. As shown in Fig. 7(d), carbon-based materials exhibit sp^2^ hybridization at 284.2 eV, and additional XPS spectra are found at 284.9, 286.1, and 290.1 eV, corresponding to C=C, C–O–C, and C=O, respectively.^41^ The V–O, Ni–O, and O–H interactions were represented by two main peaks in the spectra of O 1s (Fig. 7(e)) at 530.81 eV and 528.6 eV, respectively.^42^ Furthermore, the presence of NV–R is demonstrated by the presence of all elements of Ni^2+^ and Ni^3+^, V^5+^ and V^4+^, O, and C.

**Fig. 7:**
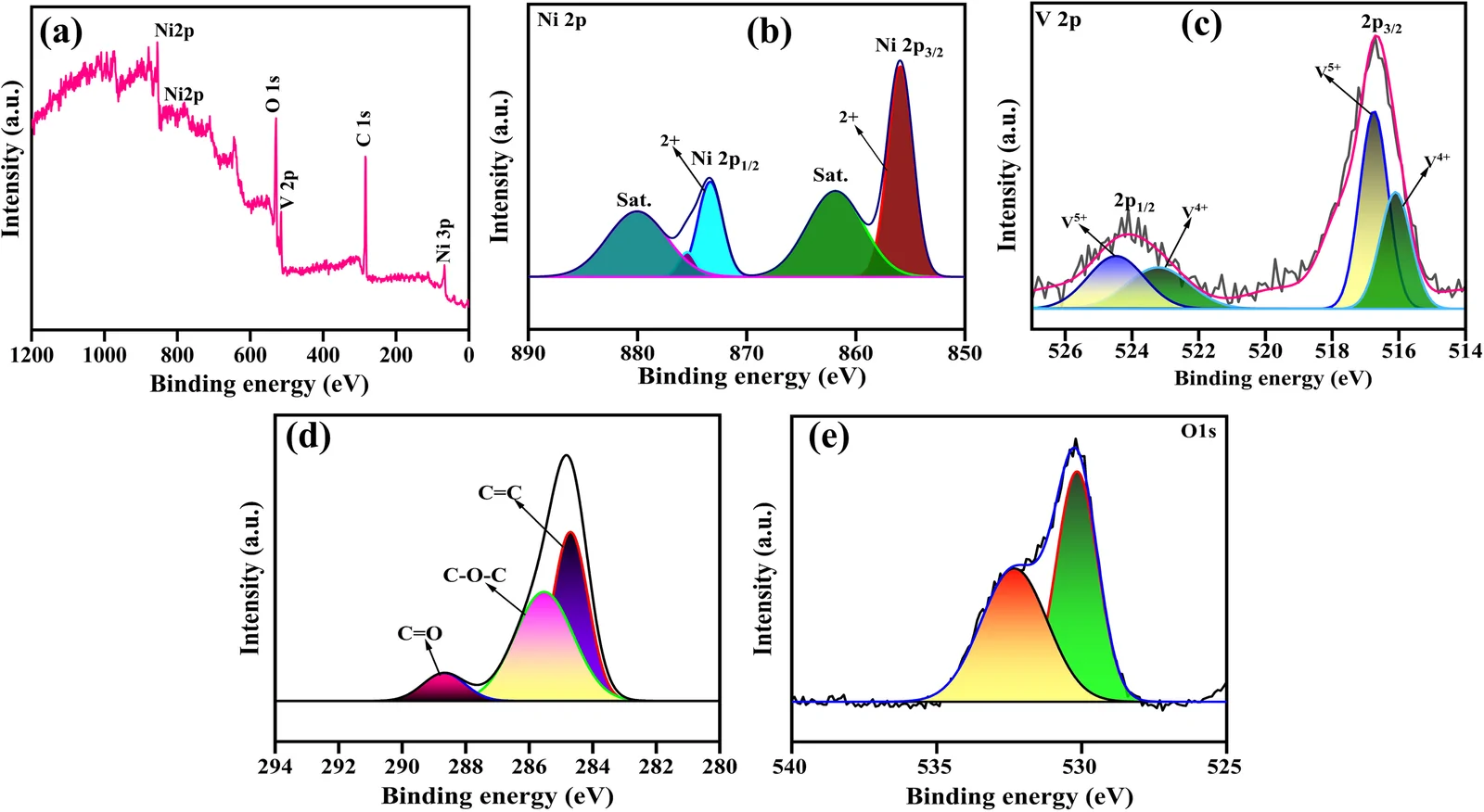
XPS spectrum of NV–R NCs, (a) Survey spectrum, (b) Ni 2p spectra, (c) V 2p spectra, (d) C 1s spectra, and (e) O 1s spectra.

### 3.5 TEM analysis

The HRTEM images are shown in Fig. 8(a and b), the lattice fringe pattern in Fig. 8(c), and the SAED pattern in Fig. 8(d). The HR-TEM images revealed that the synthesized NV–R NCs were on the layered sheets. A spherical morphology was described as being NV nanoparticles, and a thin-layered morphology as being rGO nanosheets. The *d*-spacing value is 0.415 nm, which is related to the (002) plane of rGO,^29^ consistent with the XRD assignment in Section 3.1. The SAED pattern in Fig. 8(d) of the prepared NV–R NCs also revealed a polcrystalline nature. According to XRD analysis, the ring patterns are related to the (002), (135), and (221) crystalline planes of the prepared samples.

**Fig. 8:**
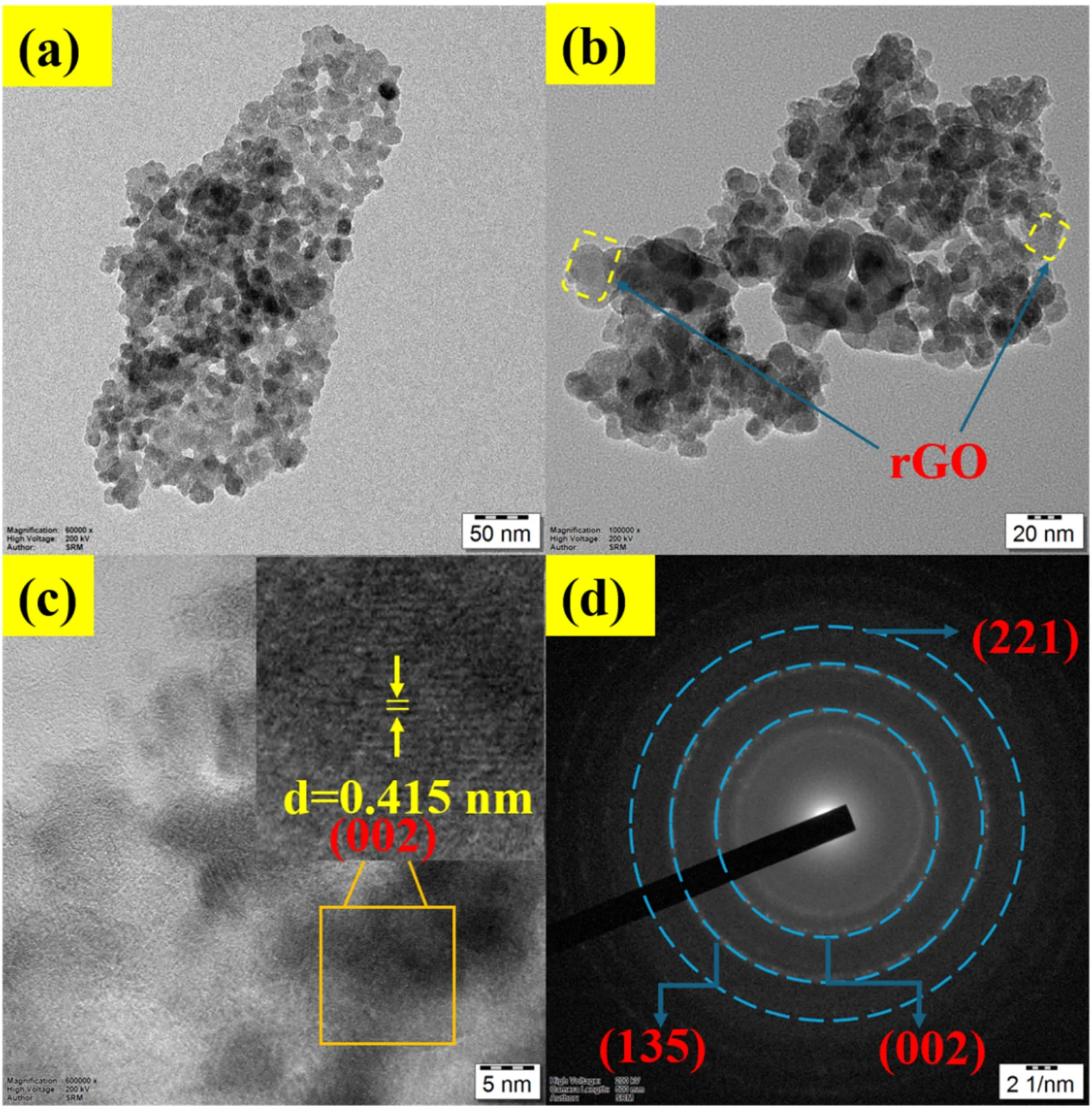
(a and b) TEM images, (c) HR-TEM images, and (d) SAED pattern of NV–R NCs.

### 3.6 Electrochemical analysis

The NV and NV–R electrodes were synthesized, and their CV analysis was performed in a 6 M KOH solution with a potential window of 0–0.5 V. The electrodes were analyzed at different scan rates from 2 to 100 mV s^−1^. The CV curves of NF, NF/rGO, NV, NV–R/PM and NV–R electrodes are given in a three-electrode configuration at a scan rate of 100 mV s^−1^, as shown in Fig. 9(a). The current response of the bare nickel foam is very small, indicating low capacitance. The current response of the NF/rGO electrode is slightly better than that of the bare electrode, because of the electric double layer capacitance of rGO. As shown for the pristine NV electrode, the presence of two redox peaks, one is the anodic and the other is cathodic, indicates the pseudocapacitive charge storage behaviour. The area of the CV enclosed in the physically mixed sample (NV–R/PM) is larger than the area in the pristine NV electrode, which indicates the positive effect of the addition of rGO, but the electrochemical response is still poor in situ-synthesized NV–R composite. It was noted that the NV–R/PM control was prepared by slurry-coating with conductive carbon black and a PVDF binder, whereas NV–R is binder-free and grown directly on the Ni foam; consequently, the performance gap between NV–R/PM and NV–R cannot be attributed to electrical contact between NV and rGO, and may also reflect differences in the electrode architecture and conductive-additive content between the two configurations. The NV–R electrode shows the highest redox current and the largest area of CV among all electrodes, which means that it has the highest charge storage capacity. This improved electrochemical performance of the NV–R electrode is consistent with improved electrical contact between Ni_3_V_2_O_8_ and rGO, which allows for faster electron transport, more electrochemically active sites and better electrolyte ion diffusion. Clear redox peaks are present in the CV analysis for the NV, NV–R/PM and NV–R electrodes, indicating that the pseudocapacitive faradaic reactions.

**Fig. 9:**
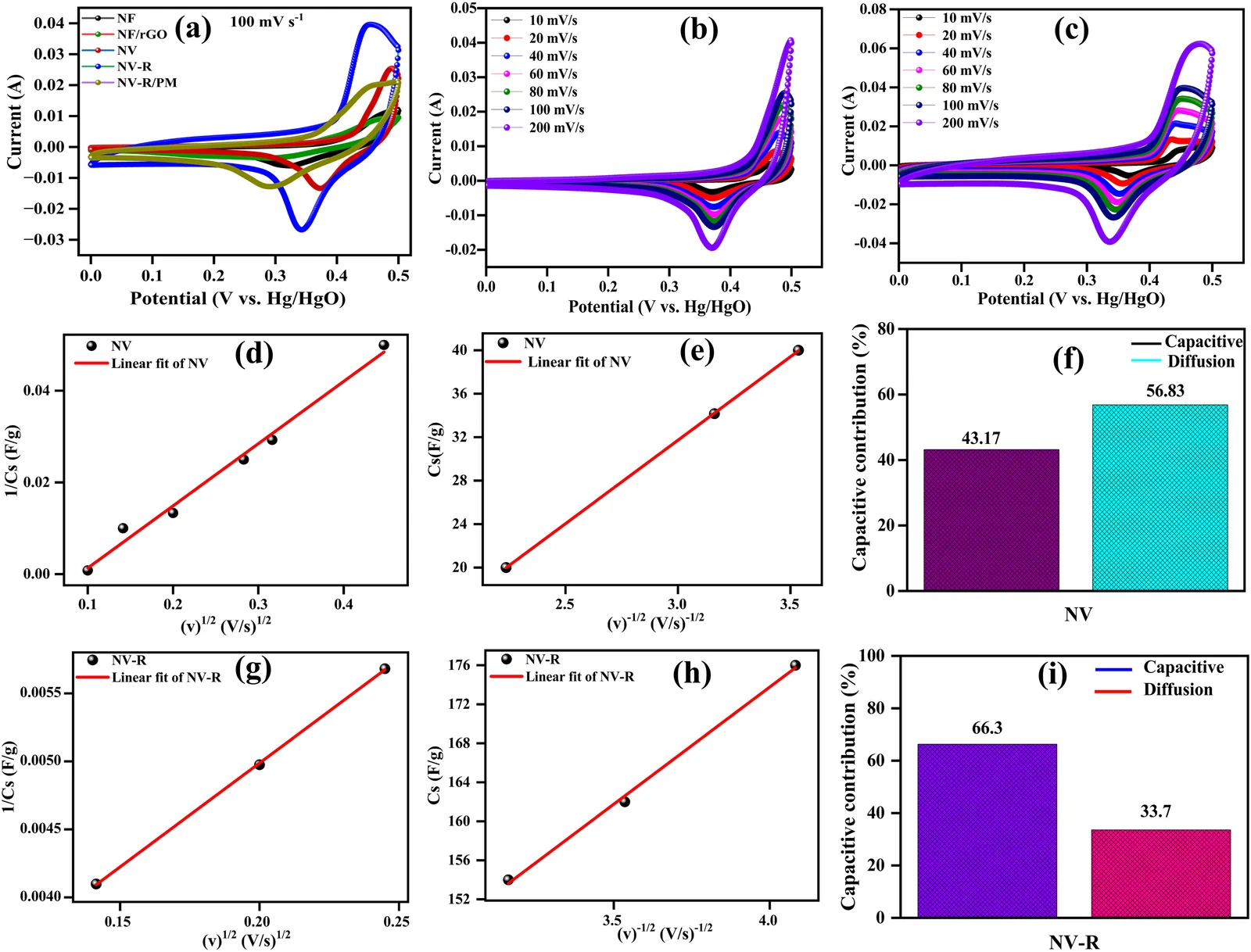
(a) CV curve comparison of NF, NF/rGO, NV, NV–R, and NV–R/PM at 100 mV s^−1^. CV analysis of (b) NV and (c) NV–R. (d) Total capacitance (*v*^1/2^*vs. q*^−1^*) and (e) outer capacitance (*v*^−1/2^vs. q*) of NV. (f) Percentage contribution of NV. (g) Total capacitance (*v*^1/2^*vs. q*^−1^*) and (h) outer capacitance (*v*^−1/2^vs. q*) of NV–R NCs. (i) Percentage contribution of NV–R by plotting the reciprocal of the specific capacity (1/*C*_s_) and calculating the square root of the scan speeds (*v*^1/2^) and total capacity of the electrode (*C*_total_).

The CV curves of the NV and NV–R electrodes obtained at different scan rates are shown in Fig. 9(b and c), respectively. The anodic and cathodic peak currents also increase with increasing scan rate, while the area of the enclosed CV increases, which means that it has a larger charge storage capacity and faster electrochemical kinetics. At higher scan rates, due to electrode polarisation and internal resistance, a slight shift of the oxidation and reduction peaks to the higher and lower potential sides, respectively.^43,44^ However, the clearly marked redox peaks remain throughout the range of scan rates and show good electrochemical reversibility and structural stability of the electrode materials. At all scan rates, the integrated CV area of the NV–R electrode is consistently larger than that of the pristine NV electrode, which shows that the NV–R electrode has a better charge-storage capability. This enhancement is due to the incorporation of rGO in the structure, which improves the electrical conductivity, allows for quick electron transport and creates effective pathways for the diffusion of electrolyte ions. Pseudocapacitive behaviour comes from reversible faradaic redox reactions of both Ni and V species. The charge-storage process is coupled with the reversible redox process of Ni^2+^/Ni^3+^ and V^4+^/V^5+^ and the adsorption/desorption of OH^−^ ions from the electrolyte. The reversible reactions are important in that they make a major contribution to the high capacitance of the NV–R electrode.^45–48^

8 Ni(OH)_2_ + OH^−^ ⇌ NiOOH + H_2_O + e^−^

9 NiOOH + H_2_O + e^−^ ⇌ Ni(OH)_2_ + OH^−^

The capacitive and diffusion-controlled charge contributions were also quantitatively evaluated *via* the Trasatti method to further elucidate the charge-storage mechanism of the NV and NV–R electrodes. This method assumes that the total stored charge (*C*_total_) is the sum of the outer surface charge (*C*_outer_), which originates from the electrode surface accessible to electrochemical reactions, and the inner surface charge (*C*_inner_), which is associated with ion diffusion into the bulk of the electrode material.^49,50^ A plot of capacitance (*C*_s_) *vs.* scan rate (*v*) is given as follows:

10 1/*C*_s_ (*V*) = const × *v*^1/2^ + 1/*C*_total_

where *C*_total_ was determined by the extrapolation of the plot of 1/*C*_s_*versus v*^1/2^ to *v* = 0. The contribution of outer and inner charges is related to the total stored charge as follows:

11 *C*_total_ = *C*_inner_ + *C*_outer_

The values of the outer surface charge (*C*_outer_) and the inner surface charge (*C*_inner_) were obtained by extrapolating the plot of *C versus v*^−1/2^ and subtracting the latter value from the former value, respectively.^51,52^ The following equation was used to obtain these contribution percentages of the inner and outer surface charges:12
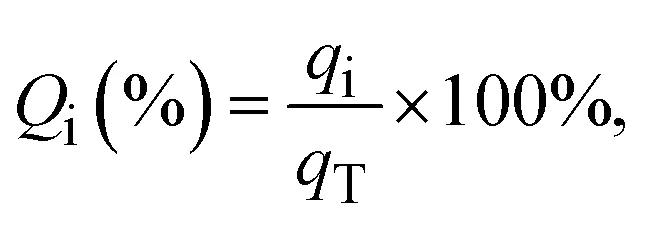
13
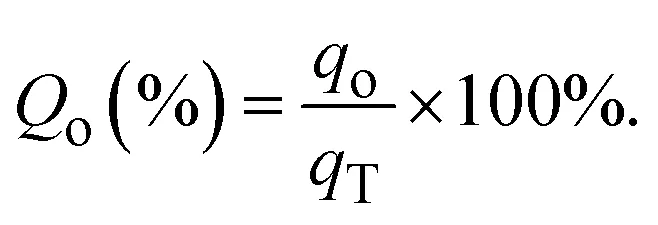


The Trasatti analysis showed that the capacitive contribution (*C*_outer_) of the NV and NV–R electrodes was 43.17% and 66.3%, respectively, whereas the diffusion-controlled contribution (*C*_inner_) was 56.12% and 33.6%, respectively (Fig. 9(d–i)). The higher capacitive contribution of the NV–R electrode indicates a larger electrochemically active surface area and enhanced charge transport, which together facilitate more efficient charge storage.^53,54^

Furthermore, the electrodes were characterized using GCD analysis in the range of 0–0.5 V (Fig. 10(a and b)). The electrodes show a non-linear shape, indicating the pseudo-capacitive nature of the electrodes. The discharge time of NV–R electrode is higher than that of the NV electrode. Moreover, the capacitance value of the electrodes was determined using eqn (2). The specific capacitance values of the NV and NV–R electrodes are 264 and 494 F g^−1^ at 1 A g^−1^, as shown in Fig. 10(c), respectively. However, the current density is increased, and the specific capacitance is decreased. At high current densities, K^+^ ions are only deposited on the electrode materials on the outside. Since K^+^ ions are inserted into the electrode materials on the inside and outside surfaces, the capacitance value is higher at lower current densities. Compared to NV, the NV–R NCs show enhanced electrochemical performance, because of their efficient synergy effect between NV and rGO.^51^

**Fig. 10:**
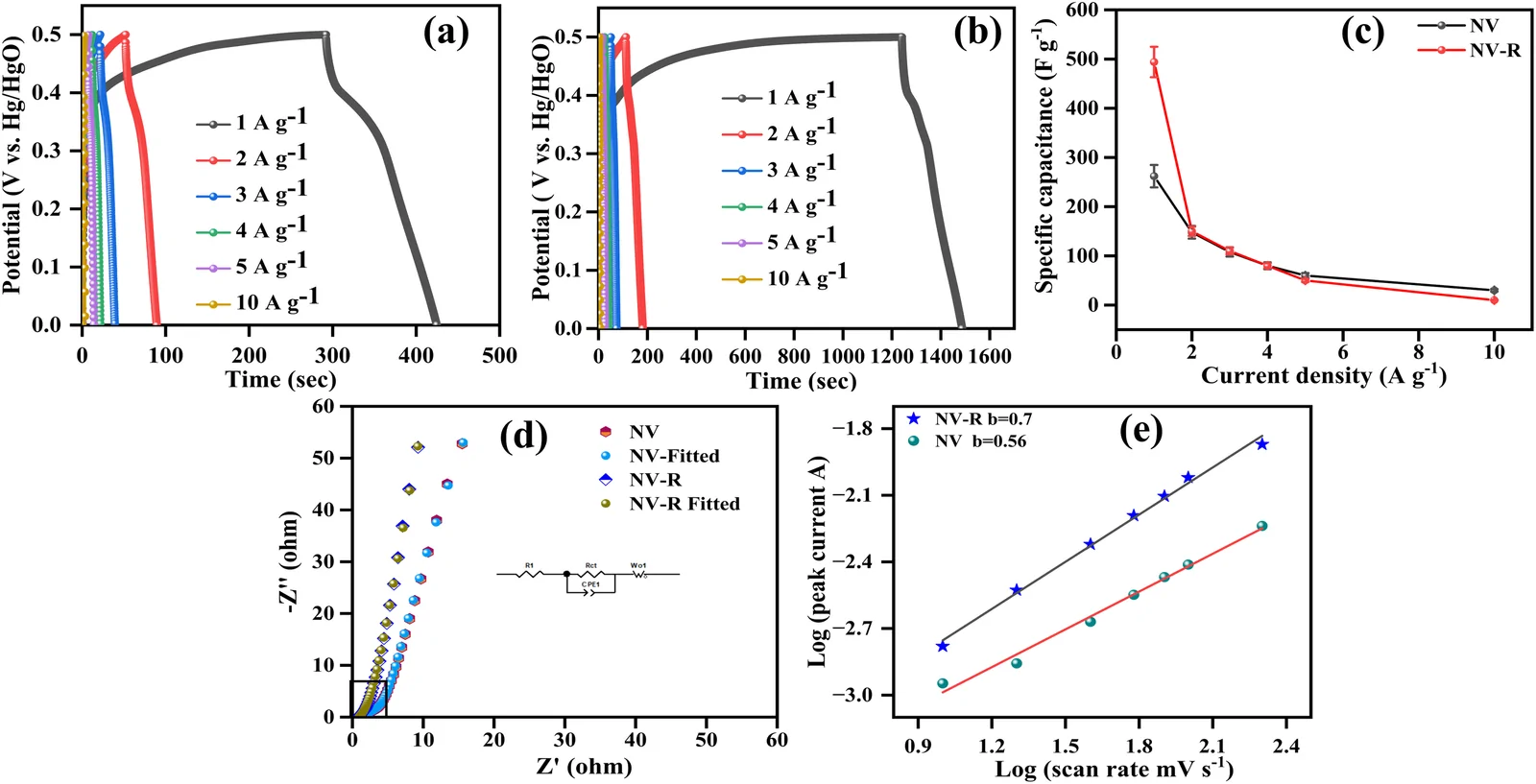
(a) GCD analysis of NV. (b) GCD analysis of NV–R. (c) Specific capacitance of NV and NV–R. (d) EIS analysis of NV and NV–R. (e) *b*-value analysis of the NV and NV–R electrodes. The results are presented as mean ± standard deviation (SD) (*n* = 3).

rGO had a specific surface area and enhanced electroconductive properties, also, the addition of rGO with the NV NPs creates an interlayer space between the rGO sheets, which facilitates electron-ion mobility and forms an electric double layer on both faces. The impedance characteristics of such nanocomposite electrodes are consistent with charge-transfer mechanisms observed in related nanosized metal oxide systems. Attaching NV NPs to the rGO surface minimizes the interaction between the nanosheets and prevents restacking and agglomeration. As a result, more accessible active sites are preserved, preventing the specific capacitance from drastically decreasing. EIS analysis was used to identify the charge transport of the prepared NV and NV–R electrodes between 100 kHz and 0.1 Hz. Fig. 10(d) illustrates the Nyquist plots of EIS for the as-synthesized NV and NV–R composite electrodes. Accordingly, the calculated *R*_ct_ values for NV and NV–R composite are 0.196 and 0.104, respectively. Compared to NV, the NV–R NCs exhibited a lower *R*_ct_ value. The improved conductivity and reduced charge-transfer resistance of NV–R are consistent with improved electrical contact between NV and rGO, together with the higher intrinsic conductivity and larger active surface area contributed by rGO. In Fig. 10(e), the charge-storage mechanism of the NV and NV–R electrodes was further examined by analyzing the charge-storage kinetics by the power law correlation between the peak current (i) and the scan rate (*v*):

14 *i* = *aν*^*b*^,

where *a* and *b* are the adjustable parameters and the value of *b* is derived from the slope of the log(*i*) *vs.* log(*v*) plot over the range of the scan rate. If the *b*-value is equal to 0.5, then the system exhibits diffusion-controlled behaviour; if the *b*-value is equal to 1.0, then it exhibits capacitive behaviour, also referred to as surface-controlled behaviour. The *b*-value of the NV electrode was 0.56, indicating that charge storage occurs mainly *via* diffusion behaviour. The NV–R electrode had a higher *b*-value of 0.70, indicating more surface-controlled/capacitive charge storage after incorporating rGO.^55^ The same qualitative trend was quantified according to the Trasatti method.

### 3.7 Asymmetric device performance

The schematic diagram of the asymmetric device is shown in Fig. 11 uses a combination AC anode and NV–R cathode to store charge. OH^−^ ions diffuse toward the NV–R surface when charged in a KOH solution. There are reversible redox reactions that mostly involve the oxidation and reduction of OH^−^ into Ni–O and V–O. When paired with ion diffusion, this method enables quick electron transport, resulting in pseudocapacitive behaviour.

**Fig. 11:**
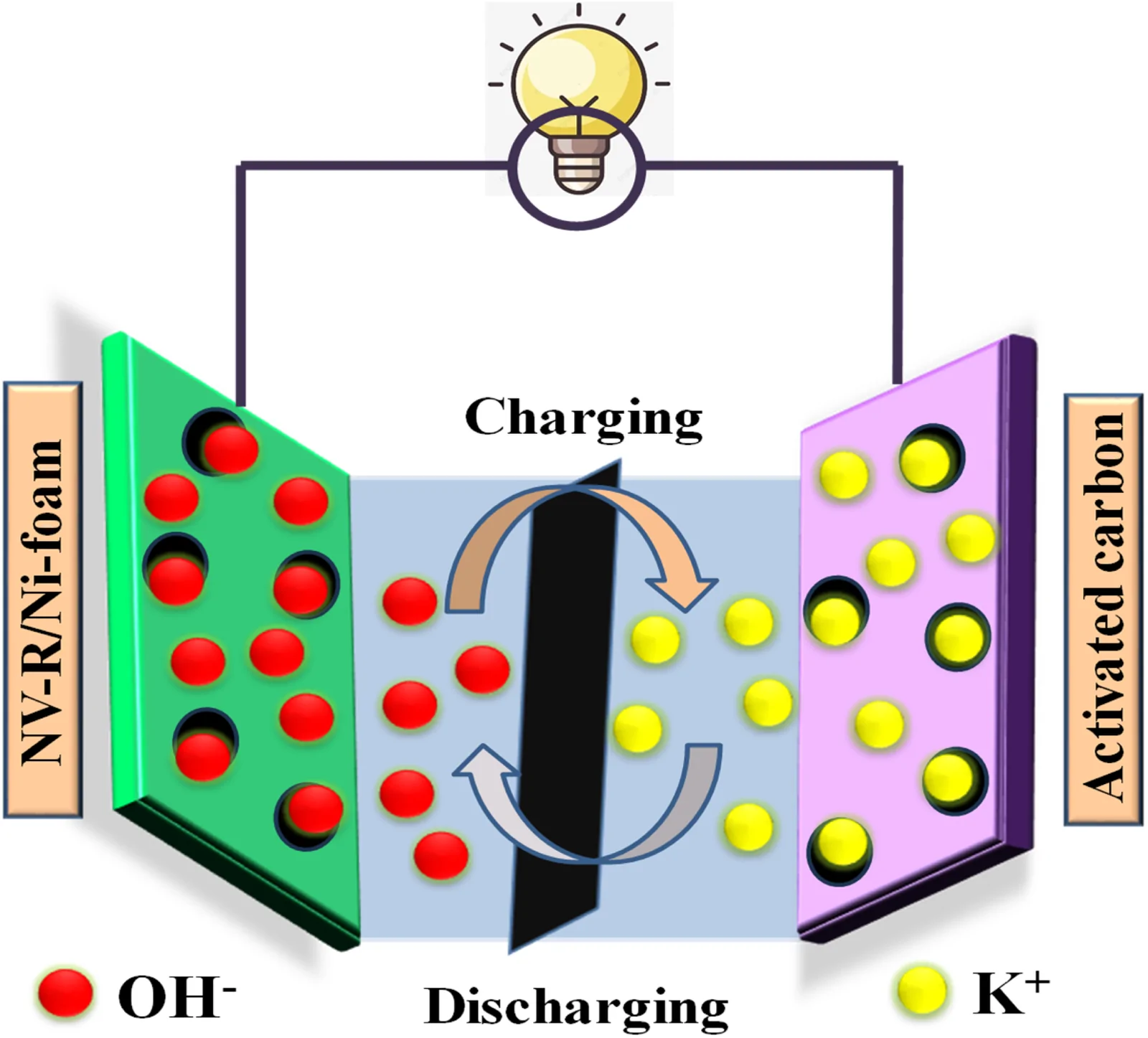
Charge storage mechanism of the NV–R ASC device.

The redox mechanism can be described as follows:

15 Ni(OH)_2_ + OH^−^ ⇌ NiOOH + H_2_O + e^−^

16 V^5+^ + e^−^ ⇌ V^4+^.

The AC electrode primarily uses non-faradaic EDLC to store charge, in which K^+^ at the electrode–electrolyte interface is adsorbed or desorbed without undergoing a chemical change. The opposite processes release the accumulated energy during discharge. Because of the asymmetric pairing, the device's potential is expanded beyond that of a single pseudo-capacitor, enabling a larger energy density. The faradaic redox activity and the fast, controlled EDLC process of NV–R results the increased capacitance, energy density, and stability. The improved electrical contact between NV and the conductive rGO network is consistent with more efficient electron transport during charging and discharging.^56^Fig. 12(a) shows the three-electrode analysis of AC and NV–R. This analysis indicated an operating potential window of 1.5 V, as shown in Fig. 12(b). The CV results of the NV–R ASC show that the redox peaks are maintained at high scan rates. The pseudocapacitive behaviour is maintained by both electrodes having easy wettability and ion diffusion pathways through the separator, which involves increased capacitance.

**Fig. 12:**
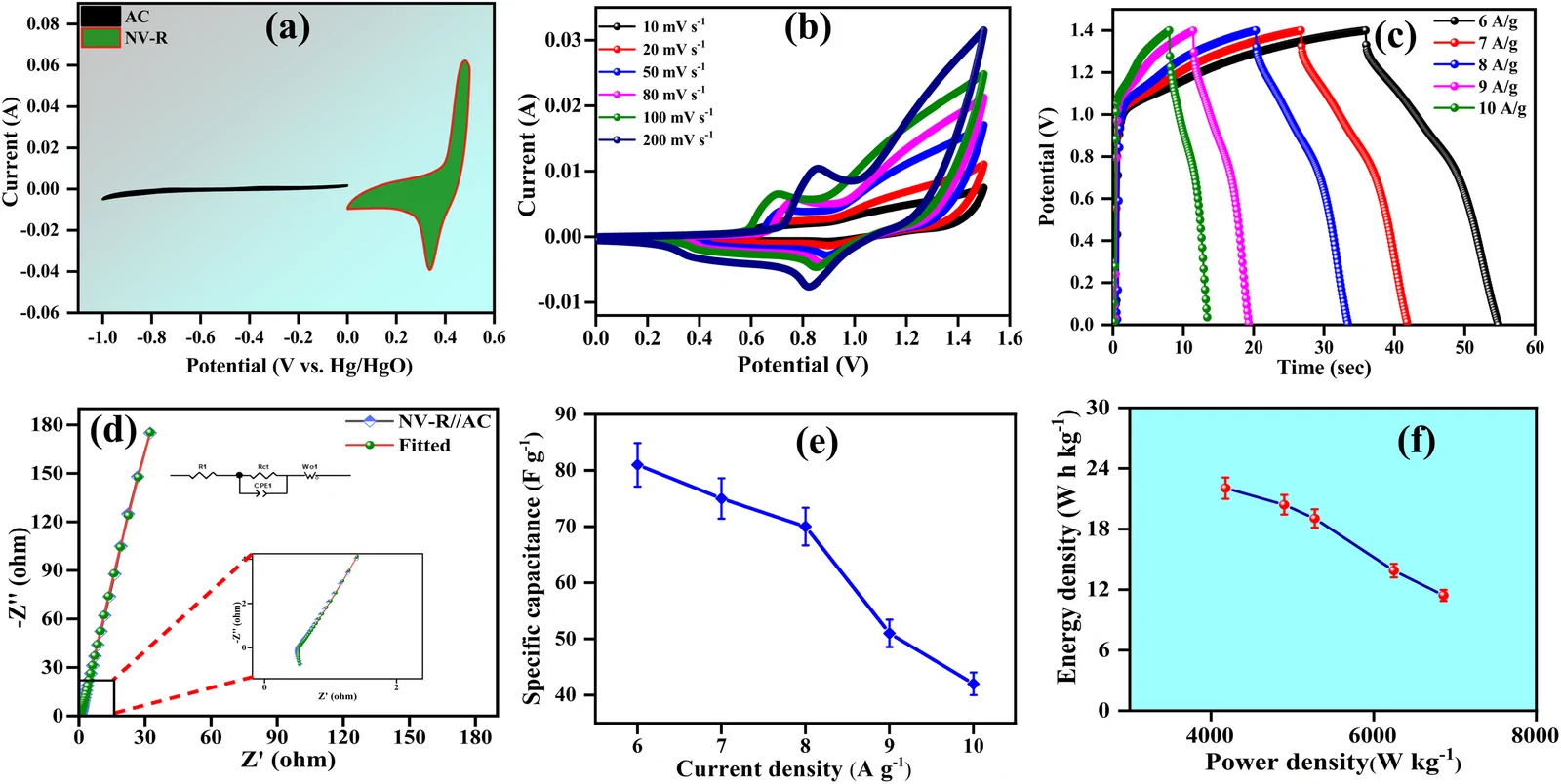
(a) CV comparison of AC and NV–R. (b) CV plots of NV–R//ASC. (c) GCD plots of NV–R ASC. (d) EIS analysis. (e) Specific capacitance of the ASC device. (f) Ragone plot of the device. The results are presented as mean ± standard deviation (SD) (*n* = 3).

The GCD curve at 1.4 V shows that the ASC has a slight IR drop, along with continued charging–discharging cycles (Fig. 12(c)). The GCD profiles of the NV–R device show asymmetric charge–discharge curves.

The EIS analysis of the NV–R ASC device is shown in Fig. 12(d). The solution resistance *R*_s_ and *R*_ct_ values were calculated, with values of 0.35 Ω and 0.52 Ω. Then, Fig. 12(e) shows the capacitance value plot of the ASC device. The capacitance values are 81, 75, 70, 51, and 42 F g^−1^ at 6, 7, 8, 9, and 10 A g^−1^, respectively. The plot in Fig. 12(f) shows an energy density of 22.05 Wh kg^−1^ at a power density of 4177 W kg^−1^. Compared with other reported vanadium-based materials, the current work shows high power and energy densities, which are presented in Table 1.

**Table 1:** Comparison of the power and energy densities of vanadium-based materials

S. no.	Asymmetric supercapacitor	Energy density (Wh kg^−1^)	Power density (W kg^−1^)	Ref.
1	Zn_3_V_2_O_8_-MWCNT//AC	12	358	57
2	Ni_3_V_2_O_8_-rGO	5.02	151.57	58
3	Ni_3_V_2_O_8_//MWCNT	4.86	1.593	59
4	Ni_3_V_2_O_8_-CNT//AC	4.55	1.6	60
5	Cu_3_V_2_O_8_@rGO	7.642	90	61
6	NV–R//AC	22.05	4177	This work

The performance of the device for practical applications was studied over 5000 GCD cycles recorded at 10 A g^−1^ for long-term cycling stability, as shown in Fig. 13. The device maintains a retention of 86.76% and an efficiency of 89.19%.^62–65^ The close physical contact between Ni_3_V_2_O_8_ and the conductive rGO network during the charge and discharge process is expected to support efficient electron transport, which helps to improve the pseudocapacitive performance of NV–R. Several factors could explain this capacity fade. The first effect is the volumetric expansion and contraction that occur with repeated insertion and extraction of OH^−^ ions in the NV lattice during continuous charge and discharge cycling, which causes mechanical stress and can cause active materials to detach from the NF current collector over time.^66^ The surface-active Ni and V species can partially dissolve or restructure during long cycling in 6 M KOH, which decreases the number of electrochemically active redox-active sites. Although rGO nanosheets are expected to help maintain the conductive network, minor restacking or agglomeration of the rGO nanosheets and NV nanoparticles at the electrode surface can also lead to a progressive decrease in active surface area during cycling. The above-mentioned results are evidence that rGO can boost the capacitance behaviour and reduce the resistivity of the NV electrode

**Fig. 13:**
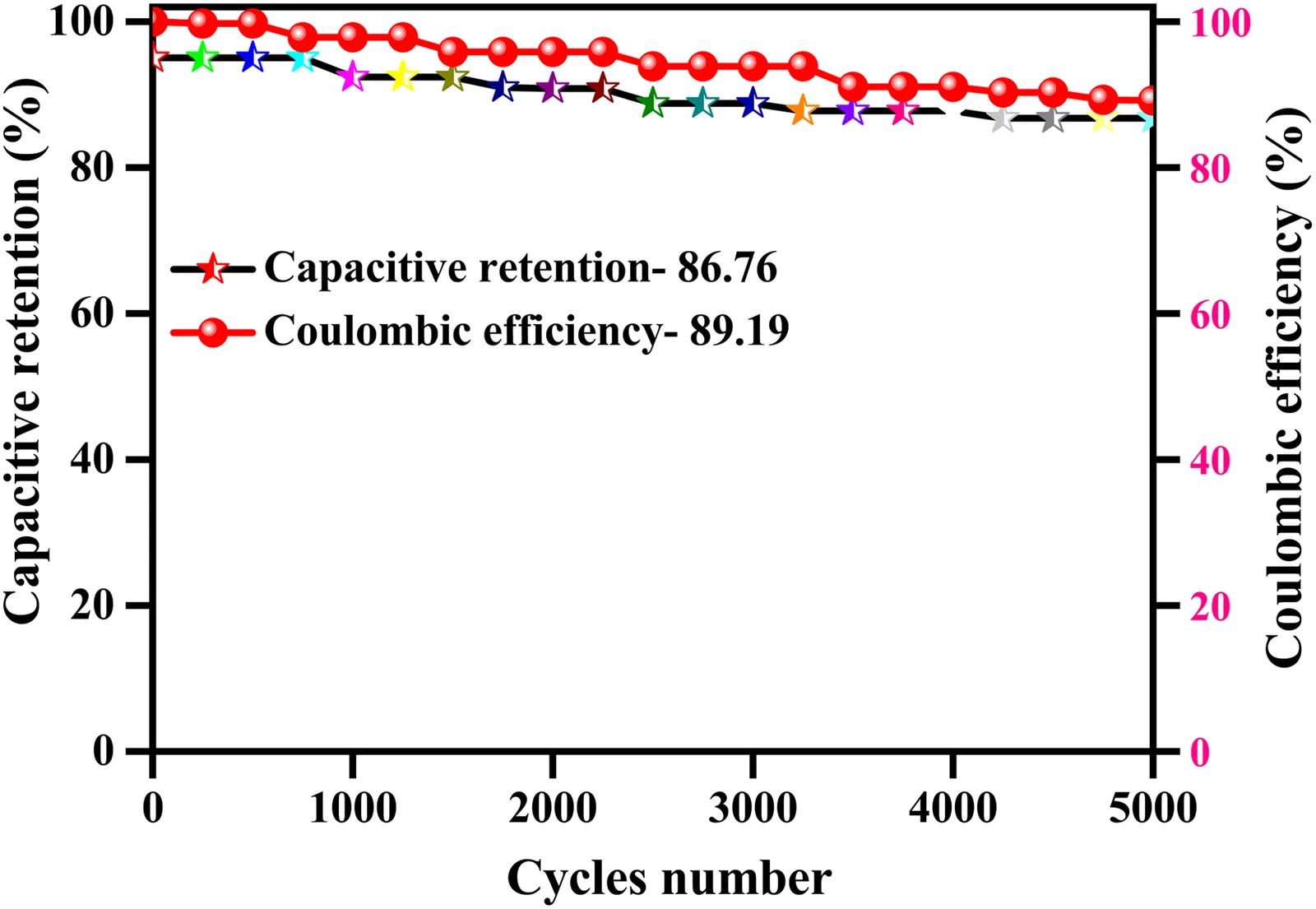
Stability and coulombic efficiency plots of the NV–R//ASC device.

## 4. Post-stability analysis

To assess the structural and morphological stability of the NV–R electrode after prolonged electrochemical cycling, XRD, SEM and Raman analyses were performed after 5000 GCD cycles, as shown in Fig. 14(a–e) The XRD pattern of the cycled NV–R electrode shown in Fig. 14(a) still exhibited the characteristic diffraction peaks of Ni_3_V_2_O_8_, while the extra peaks indicated in the triangles were due to the underlying Ni-foam substrate and not the active material. No impurity phase or structural decomposition was detected. The Raman spectra taken before and after cycling are presented in Fig. 14(b). The characteristic Raman features of Ni_3_V_2_O_8_ persisted following cycling, but the Raman spectra had lower peak intensities, higher background, and higher spectral noise, which were attributed to surface disorder and partial amorphization of the near surface region and possible electrolyte residues that had collected during cycling. No other bands than those from the secondary phases were found. The intensities of the relative peaks are slightly different, but this is within the range of variation caused by surface roughening and nanoparticle agglomeration, as observed by SEM (Fig. 14(c–e)) and is not considered to be a bulk phase transformation; therefore, the crystal structure of NV–R does not change after repeated charge/discharge cycling. The SEM images also confirm this morphological stability. The NV–R electrode had a well-defined nanostructure prior to the application of cycling. The overall morphology was mostly retained following the stability test (Fig. 14(d and e)), but some surface roughening and nanoparticle aggregation were observed.

**Fig. 14:**
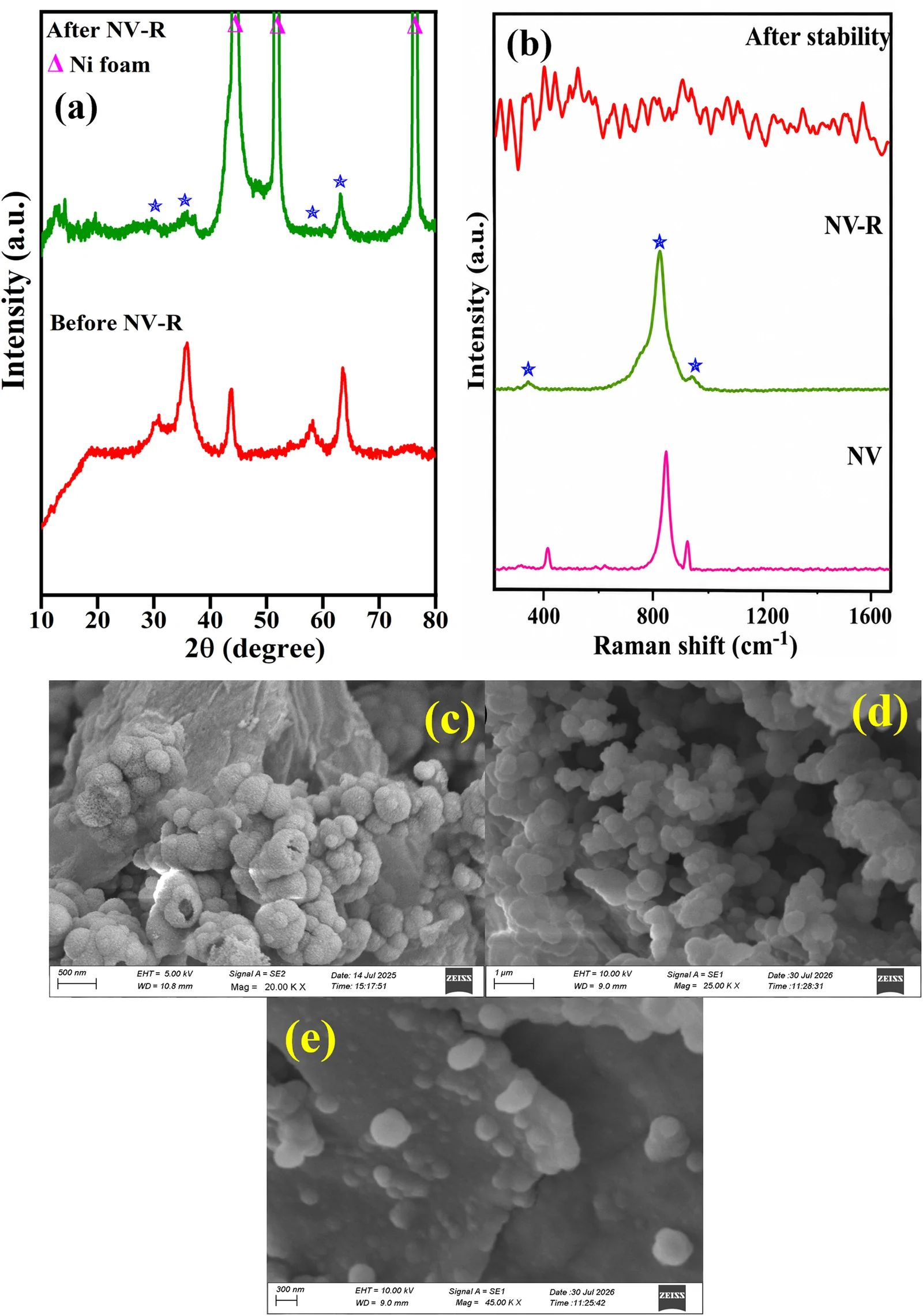
(a) XRD profiles of NV–R before and after stability testing, and (b) Raman spectra of NV–R before and after stability testing. SEM images (c) before and (d and e) after prolonged cycling under different magnifications.

No delamination from the substrate and no structural collapse were observed during cycling, which shows that the structure of the electrode was mechanically stable during cycling. These results, coupled with the XRD and SEM results, validate that the crystal structure of NV–R is preserved and only surface properties are altered, leading to the observed slight loss of signal quality for all three methods. It should be noted that a pristine NV electrode subjected to the same 5000-cycle was not characterized here; therefore, the above XRD, SEM and Raman results demonstrate the post-cycling structural and morphological stability of the NV–R electrode itself, rather than a direct, cycle-matched comparison, establishing that rGO suppresses structural degradation relative to pristine NV.

## 5. Conclusion

In summary, NV and NV–R NCs were prepared using a simple hydrothermal method, and detailed physicochemical and electrochemical characterization was performed. The addition of rGO nanosheets resulted in good supercapacitor performance, due to its high electrical conductivity, rapid electron/ion transport and electrochemically active sites and also significantly enhanced the specific capacitance of Ni_3_V_2_O_8_, with the NV–R electrode achieving 494 F g^−1^ compared to 264 F g^−1^ for pristine NV, respectively. Additionally, the fabricated asymmetric supercapacitor showed a maximum energy density of 22.05 Wh kg^−1^ at a power density of 4177 W kg^−1^, which is a good energy storage capability. The NV–R electrode also exhibited a good cycling stability, maintaining 86.76% of its initial capacitance after 5000 charge–discharge cycles. The NV–R electrode exhibited low solution resistance *R*_s_ and *R*_ct_ values of 0.35 Ω and 0.52 Ω, respectively, suggesting good charge transport properties and favourable electrochemical reaction kinetics. In summary, these results confirm that Ni_3_V_2_O_8_ and rGO exhibit a synergistic effect, making the NV–R nanocomposite a promising electrode material for high-performance supercapacitor applications.

## Declaration of generative AI and AI-assisted technologies in the manuscript preparation process

During the preparation of this work, the author(s) did not use any AI tools or services.

## Author contributions

B. Kabilan, Manikandan Ayyar, S. Sakthivel, and R. P. Patil: conceptualization, data curation, formal analysis, funding acquisition, investigation, methodology, project administration, resources, software, supervision, validation, visualization, writing – original draft, and writing–review and editing. V. Mohanavel and Saravanan Rajendran: data curation, formal analysis, investigation, methodology, validation, visualization, writing – original draft, and writing–review and editing. D. Shanmugapriya: validation, visualization, and writing–review and editing. All authors have read and agreed to the published version of the manuscript. Tahseen Kamal: writing–review and editing. Anish Khan: writing–review and editing. Hadi M. Marwani: writing–review and editing. Yasmeen G. Abou El-Reash: writing–review and editing, Funding. Hany Koheil: writing–review and editing, Funding. Basem E. Keshta: writing–review and editing.

## Conflicts of interest

The authors declare no competing interests.

## Data Availability

The datasets generated and/or analysed during the current study are available from the corresponding author upon reasonable request.
